# Involving Children and Teenagers With Bilateral Cochlear Implants in the Design of the BEARS (Both EARS) Virtual Reality Training Suite Improves Personalization

**DOI:** 10.3389/fdgth.2021.759723

**Published:** 2021-11-12

**Authors:** Deborah Vickers, Marina Salorio-Corbetto, Sandra Driver, Christine Rocca, Yuli Levtov, Kevin Sum, Bhavisha Parmar, Giorgos Dritsakis, Jordi Albanell Flores, Dan Jiang, Merle Mahon, Frances Early, Nejra Van Zalk, Lorenzo Picinali

**Affiliations:** ^1^Sound Laboratory, Cambridge Hearing Group, Clinical Neurosciences, University of Cambridge, Cambridge, United Kingdom; ^2^St Thomas' Hearing Implant Centre, Guys and St Thomas' NHS Foundation Trust, London, United Kingdom; ^3^Reactify Music, London, United Kingdom; ^4^Audio Experience Design, Dyson School of Design Engineering, Imperial College London, London, United Kingdom; ^5^Psychology and Language Sciences, Faculty of Brain Sciences, University College London, London, United Kingdom; ^6^Department of Respiratory Medicine, Cambridge University Hospital NHS Foundation Trust, Cambridge, United Kingdom; ^7^Design Psychology Lab, Dyson School of Design Engineering, Imperial College London, London, United Kingdom

**Keywords:** spatial hearing, bilateral, cochlear implant, virtual reality, training, action research, participatory design, children

## Abstract

Older children and teenagers with bilateral cochlear implants often have poor spatial hearing because they cannot fuse sounds from the two ears. This deficit jeopardizes speech and language development, education, and social well-being. The lack of protocols for fitting bilateral cochlear implants and resources for spatial-hearing training contribute to these difficulties. Spatial hearing develops with bilateral experience. A large body of research demonstrates that sound localisation can improve with training, underpinned by plasticity-driven changes in the auditory pathways. Generalizing training to non-trained auditory skills is best achieved by using a multi-modal (audio-visual) implementation and multi-domain training tasks (localisation, speech-in-noise, and spatial music). The goal of this work was to develop a package of virtual-reality games (BEARS, Both EARS) to train spatial hearing in young people (8–16 years) with bilateral cochlear implants using an action-research protocol. The action research protocol used formalized cycles for participants to trial aspects of the BEARS suite, reflect on their experiences, and in turn inform changes in the game implementations. This participatory design used the stakeholder participants as co-creators. The cycles for each of the three domains (localisation, spatial speech-in-noise, and spatial music) were customized to focus on the elements that the stakeholder participants considered important. The participants agreed that the final games were appropriate and ready to be used by patients. The main areas of modification were: the variety of immersive scenarios to cover age range and interests, the number of levels of complexity to ensure small improvements were measurable, feedback, and reward schemes to ensure positive reinforcement, and an additional implementation on an iPad for those who had difficulties with the headsets due to age or balance issues. The effectiveness of the BEARS training suite will be evaluated in a large-scale clinical trial to determine if using the games lead to improvements in speech-in-noise, quality of life, perceived benefit, and cost utility. Such interventions allow patients to take control of their own management reducing the reliance on outpatient-based rehabilitation. For young people, a virtual-reality implementation is more engaging than traditional rehabilitation methods, and the participatory design used here has ensured that the BEARS games are relevant.

## Introduction

Advances in mobile technologies have resulted in the development of flexible platforms providing personalized interventions that enable patients to take control of their own health care.

In recent years, the importance of involving patients in the development of clinical interventions has become apparent to maximize engagement, to improve usability and potential success, and more importantly to ensure that the intervention is ultimately relevant for the targeted patient group (1).

Hakobyan et al. (2) highlighted the importance of incorporating patient groups in the design of mobile technology-based interventions to ensure that they meet the needs of the specific population. Participatory design recognizes and involves the key stakeholders in the design and development of the intervention. Without such input, historically, information technologies typically only achieve 40% of population engagement with the intervention, as reported by Hakobyan et al. (2). In spite of this, most interventions still do not involve patients in the development phase. From 18 articles describing the development of mobile technologies reviewed by Hakobyan et al. (2), only four incorporated quality participatory design in the process.

Here we have used participatory design for the development of a virtual reality training suite for improving spatial hearing for 8–16 year-olds with bilateral cochlear implants (CI). This training suite is called BEARS (Both EARS). The development of the BEARS training suite is driven by the fact that normal-hearing listeners use subtle differences in timing and level of sounds reaching each ear to provide directional cues (3–6) that help to separate speech from noise and the ability to attend to a particular speaker (7–9). Although language development, sound localization, speech-in-noise perception and listening effort are better for people with bilateral cochlear implants compared to those with a unilateral implant, these skills remain far below those of normally-hearing children (10–18). Neural plasticity exists for spatial hearing improvements through training (19). Improvements are driven by two processes: (1) cue remapping (the use of new spatial cues to construct a new localisation map, most likely the use of monaural frequency cues in the unprocessed ear), and (2) cue reweighting (the reliance on any unaltered cues while ignoring the altered ones) (20–23). Evidence about these processes is found in several reports. For instance, listeners can adapt to changes in spectral cues, which are critical for judging elevation, as well as solving front-back confusions, even when these are altered (24–26). There has been some discussion about what type of training is most appropriate for feasible delivery and maximization of any benefits. Computer-based training has great potential as it can be delivered anywhere without requiring a face-to-face appointment, and is engaging for most people, especially for children and teenagers. It has been shown that computer-based training can improve speech-in-noise perception for people with cochlear implants (27–30). Green et al. (31) observed an average 2 dB improvement in speech reception thresholds for sentence recognition in babble after 12 h of computer-based training. The nature of the training stimuli has also been explored by previous research. Cai et al. (32) found that audio-visual training is more effective than auditory-only training, and Steadman et al. (23) outlined the importance of auditory-based interaction during the training. A systematic review by Rayes et al. (33) that looked at the effectiveness of training in children with CIs found that the most effective intervention involves the use of multiple modalities or a combination of bottom-up and top-down training tasks. Finally, Whitton et al. (34) explored the transfer and generalization of the acquired training, outlining that audio-motor perceptual training can enhance speech in noise intelligibility by up to 25%.

Based on the evidence summarized above, we designed and carried out the research reported here. The objective was to collaboratively design and develop a training intervention for young people with bilateral cochlear implants, aiming at improving their listening skills, and specifically focusing on the spatial sound cues provided by their cochlear implants.

## Methods

An action research study design was employed (35, 36). Within action research, development and change are achieved through the simultaneous process of taking action whilst conducting research, all informed by user involvement and governed by critical reflection. All stakeholders and researchers are equal members of the research team. Within this study, three phases were employed in the process of development of the BEARS training suite, to ensure that it is appropriate for the intended population.

**Figure 1:**
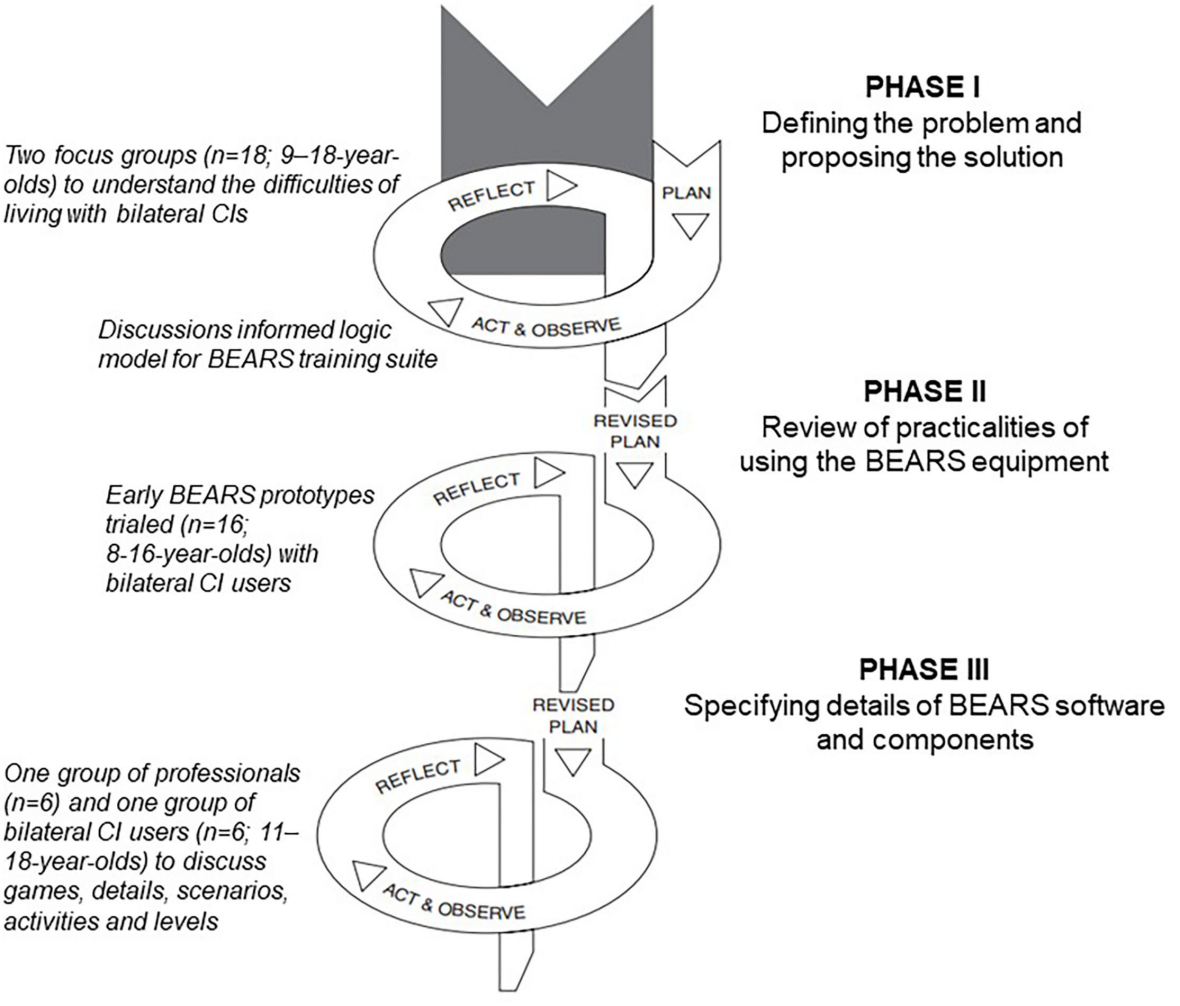
The cycles for the three participatory design phases.

The stakeholders involved in the process were: young people and young adults using bilateral CIs, family and friends, teachers, engineers and developers, speech and language therapists, music therapists, and audiologists. The process involved multiple focus groups for feedback, reflection, and critical appraisal, each of which was run by an independent facilitator. In advance of each focus group, the goals and topic guides were developed amongst the research team and summary notes were produced for each meeting. Where the meetings were broken down into discussion groups, a note taker and facilitator were assigned to each group. The feedback was reviewed after the focus groups and a plan, consisting of a set of actions, was created and prioritized for the next stages of action and implementation. See Figure 1 for an outline of the three phases.

Phase I—defining the problem and proposing the solution. Facilitated in-person discussions were conducted with two focus groups (*n* = 18) of CI users aged between 9 and 18 years of age. One group consisted of 10 participants (*n* = 6 male, *n* = 4 female) at a school for deaf and hard-of-hearing (DHH) children, who volunteered to help in response to an advert. The other eight (*n* = 3 male, *n* = 5 female) were recruited from an advert circulated by a charity and attended a meeting held in London.

The goal of these meetings was to understand the difficulties of living with bilateral CIs. The issues were discussed by the two groups and prioritized in terms of overcoming the difficulties discussed to understand what the acceptable interventions were. Based on the discussions, a logic model was created by the research team to underpin the planned multi-modal BEARS virtual reality auditory training intervention.

Phase II—review of the practicalities of using the BEARS equipment. The first implementations of BEARS were trialed by 16 children aged 8–16 years. This group was made up of from older children and teenagers from mainstream and special schools (*n* = 6 male, *n* = 5 female) with an additional 5 younger participants (*n* = 2 male, *n* = 3 female) to help determine if the BEARS training suite would be appropriate for a wider age range of CI users. The goals of the cycles that made up phase II were to understand the practical limitations of BEARS with respect to how frequently training should be conducted, whether the head-mounted display worked well for all listeners, and what sort of age adaptations were required. This phase involved two cycles of action and reflection. In the first cycle, participants were given the BEARS headsets with one game to provide feedback on ease of use. In the second cycle, we held an in-person event with multiple stations for participants to visit to give feedback on different aspects that had been developed and to discuss these with their peers.

Phase III—definition of the details of the BEARS software tools and components. This phase was conducted with two groups. The first were adult professionals working across CI Centres and in the local educational support services. This included teachers of the deaf, speech and language therapists, and audiologists (*n* = 6). They reviewed the tools from a clinical and educational perspective. The second group were bilateral CI users (aged 11–18 years; *n* = 3 male, *n* = 3 female).

Due to the COVID pandemic, the groups in phase III were conducted virtually using Microsoft Teams. For the teachers and clinicians, the entire group discussed the different software tools together. For the bilateral CI group, pre-allocated breakout rooms were set up to discuss the different elements of the applications. Each group was made up of two bilateral CI users who were matched to work well together (based on teacher opinion), one facilitator and one notetaker.

## Results

### Phase I—Defining the Problem and Proposing the Solution

The CI focus groups were asked to discuss freely about their listening difficulties that they faced in everyday life, what they thought might cause or have an effect on these, and any activities (interventions) that they had found helpful. The notes on the difficulties were fed back to the groups and they grouped points together and set priorities for the project.

The combined prioritized statements from the two groups were:

1) Everyday listening requires “extra effort” which makes communication “tiring” and ultimately “challenging.” These problems were particularly reported with respect to noisy environments.
2) It can be difficult to “combine” sounds from two CIs because the two ears often do not sound the same. Sequential implantees (>1 year between two implants) reported that second CI could seem “annoying,” “distracting,” and “lop-sided,” which in some cases resulted in non-use.
3) Listening training can be helpful but current rehabilitation techniques are not always engaging, and relevant, and computer-based approaches may be more motivating.

Based on the feedback, the research team reviewed the information, discussed ideas for addressing the issues and decided to develop the BEARS training suite to address the issues. The research team created goals that the BEARS training suite needed to meet. These goals were:

1) To include age-appropriate and engaging listening games.
2) To use multiple training tasks (speech in noise, localisation, and music) to optimize effectiveness
3) To use visual cues to support engagement
4) To implement in virtual reality to enhance the gaming aspect
5) To use gaming head mounted display headsets for flexibility and usability
6) To develop the game soundscapes using the established 3D Tune-In toolbox (37).

The research team developed the following logic model to underpin the BEARS intervention. The logic model was developed following the UKs Medical Research Council and National Institute of Health and Care Excellence (38) advice that the development of new interventions should outline the mechanism of change for an intervention. A logic model outlines the theory of how an intervention will lead to the desired outcomes:

*People with bilateral CIs struggle to understand speech in noise, making communication tiring and challenging, having a negative effect on social integration and well-being. The problem underpinning this is that bilateral CI users are not effectively using the cues from both ears to maximise spatial hearing. The use of the BEARS training suite with audio-visual information and multiple listening modalities (speech in noise, localisation, and music) should improve spatial hearing, speech-in-noise perception and ease of listening. The change mechanisms for these effects will be plasticity-driven processes enhancing learning and maximising spatial listening skills performance. Factors supporting these training-induced effects are audio-visual integration, multimodal stimulation, and cognitive engagement that drives generalisation to other auditory stimuli. These changes will lead to better communication and social engagement skills (with reduced fear of embarrassment) which in turn will improve quality of life through healthier social and emotional-regulation development. As communication becomes easier, self-confidence continues to build up, improving development in multiple areas of life such as building relationships and education*.

*Motivation, engagement, rewards, time commitment, and developmental processes will act as modulators. Behaviour changes will also increase uptake and usability*.

**Figure 2:**
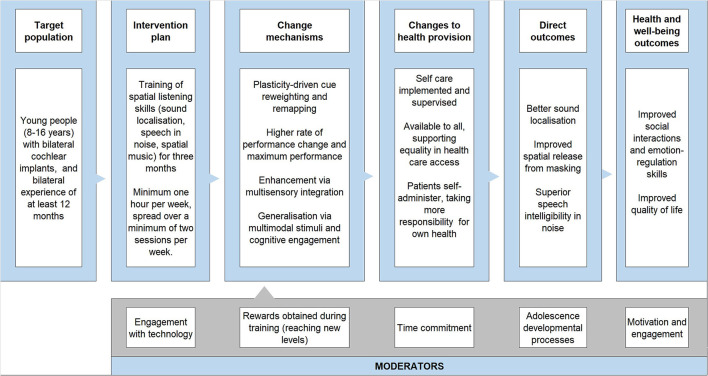
The logic model to explain the BEARS training suite rationale.

See Figure 2 for schematic of logic model.

The target population is older children and teenagers (aged 11–16 years) with at least 12 months of bilateral CI experience rather than newly implanted bilateral users. This therefore precludes influencing post-activation plasticity-driven processes for developing spatial listening skills. Plasticity is assumed to be ongoing even after 12 months of usage, as research indicates that the associations between learning and cognitive control performance only emerges with age and appears most prominent for late adolescents (39). Studying bilateral CI users 12 months after implantation allows for the effects of habituation, as they would have grown accustomed to wearing and interacting with their implants, thereby minimizing the confounding effects of novelty and/or any issues arising from initial use. Also, the numbers of older children and teenagers who receive bilateral cochlear implants are not large. The patient groups who reported difficulties had typically received their implants as young children, so this is the population who are focussed on initially but the work can be applied to other groups.

### Phase II—Practicalities of Using BEARS

The feedback from the first cycle of phase II indicated for 67% (10 out of 15) of the CI users that the use of the head-mounted display with headphones was practical. The remaining 33% of the CI users found the systems either too bulky (three people) or had balance issues because of eyes being covered with the virtual reality goggles (two people). The comments about bulkiness came from young people aged 8–10 years who were younger and smaller than the majority of the participants.

All participants reported that they found the games enjoyable and the instructions were straightforward. They requested that the differences between levels were made smaller. The initial game that was trialed was aimed at improving localisation ability, and its effectiveness had already been validated in previous work (23). In the game, the goal was to identify the position of an audio-emitting alien ball and shoot at it. In the lower levels there were audio and visual cues but in the higher levels there were just audio cues and they found the move to sound alone too sudden. They also requested more rewards and the ability to play the game online with friends. Parents liked that the young people were able to play the games independently.

The research team reviewed the feedback and implemented an option for an iPad-based game to avoid wearing bulky equipment or covering eyes. A greater number of levels were introduced so that the visual cues were faded out more gradually and positive feedback was added in.

It was not possible to make the game interactive with other online players or to allow an online scoresheet comparison because the BEARS training suite will be evaluated in a clinical trial and this sort of engagement with others might contaminate the trial. However, the wish for a BEARS users' community has been recorded for future implementations.

In the second cycle of phase II, the participants attended a group event and visited the aforementioned stations to provide ideas for how to expand and implement new games that would be engaging for training speech-in-noise perception and music. For speech-in-noise training, the groups suggested having café scenarios where the user has to listen out for different food/drink orders or key words given by customers presented at different locations. The complexity of the game would change based on the range of locations or background noise. For music training, they recommended a game where one can make music by drawing in different musical instruments and adjust the sound of the instruments. They also liked the idea of identifying and discriminating different songs. The final idea that they wanted to incorporate was to identify when a specific instrument was present and the complexity would be built up by adding in other musical instruments and moving the location which could also be identified.

The group had ideas for making the localisation training game more appropriate for the younger group by catching butterflies or popping bubbles.

The research team were able to incorporate all of these suggestions into initial game prototypes for the three training tasks. These took the form of:

1. A localisation training game, involving identifying the position of an audio-emitting alien ball and shooting it. The ball is visible only in the early levels, and gradually disappears as soon as the player advances.
2. A spatial speech-in-noise recognition training game, involving listening to and identifying customer food/drink orders and serving the correct item.
3. A music training game, involving completing a number of music-oriented challenges to progress through an escape room.

The games were then reviewed in phase III. It was decided that having realistic lip-syncing for the speech-in-noise games was not practical and that the characterisation would be more appropriate as cartoon characters than realistic/natural appearance.

### Phase III—Definition of the Details of the BEARS Software Tools and Components

**Figure 3:**
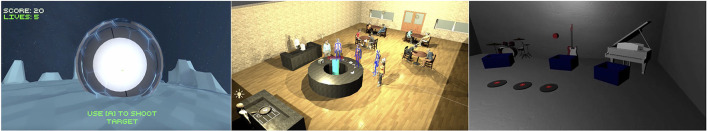
(Left) Localisation game. (Middle) Speech-in-noise game. (Right) Music training game.

For phase III, video demonstrations of the games were prepared to show to the participants in the online groups. All participants had the opportunity to review the videos in advance (see Figure 3) for images of the 3 different games themes. For the first group, the clinicians gave feedback on how to maximize speech and language development with the games and gave ideas for vocabulary to use in café scenes to provide minimal contrasts for example “peas” vs. “cheese.” They also suggested changing the carrier phrases to ensure that the contrastive word did not always fall at the end. They also recommended a shop as an alternative to a café.

The young participants reviewed the appearance of the avatars, provided their opinions, and voted on the preferred style to use. They also recommended adding in a wildlife park scenario to increase engagement and interest for a wider age range of listeners. For the music games, they recommended additional artists that would be more appropriate to include.

Note that in the next iteration, phase III will have an additional face-to-face cycle to verify the final version of the games and the implementation using the latest hardware (the initial equipment review was conducted three years ago). We will also verify that the 8–10 year-olds are happy with the modifications that were implemented for their age group. At that stage the BEARS training suite will be finalized for use in a clinical trial.

## Discussion

We have outlined the formalized participatory design approach that was used to develop the BEARS training suite, based on multiple action research cycles.

In spite of their potential to maximize patients' adoption of new technologies for delivering training, formalized participatory designs have not been extensively reported in the field of hearing research. There are only a few articles reporting research with young people (older children and teenagers). For instance, Hallewell et al. (40), already mentioned, and Hanssen and Dahl (41), who used participatory design in the development of an interactive sound environment simulator to facilitate communication between audiologists and patients. The authors promoted the value of participatory design to maximize effectiveness of complex interventions that affect both patients and practitioners. Ferguson et al. (42) worked closely with clinicians and hearing-aid users to develop the content and delivery approach for a series of video tutorials that support first-time hearing-aid users. Frost et al. (43) developed an auditory-cognitive training application which was intended to delay the onset of dementia. Their stakeholders were clinicians from audiology and cognitive disorders specialties. All of these reports highlight the importance and value of involving patients and clinicians together to maximize the effectiveness of new hearing healthcare interventions.

BEARS is considered to be a complex intervention because of the multiple training elements within the package and the possibility for tailoring the intervention for individual needs, for instance, those arising from factors such as age.

As a complex intervention it is recommended by the MRC-NIHR (38), guidance that the development phase should have a clearly stated outcome that the intervention should achieve. The outcomes were determined and prioritized by young bilateral CI users to be an improvement in speech-in-noise perception such that not only lead to improved accuracy but also that the level of listening effort is reduced. Based on theoretical and clinical knowledge, a logic model was developed to define the likely mechanism for change for the use of the BEARS training suite.

Many stakeholders were involved in this development phase and the approach for running the different focus groups had to be adapted to be appropriate for the participants themselves. We separated out the professionals from the young bilateral CI users. In addition, we separated out the young bilateral CI users into those at primary school (8–11) and those at secondary school (11–16). The purpose of the separation of the different groups was to ensure that all participants felt comfortable to engage and contribute to the discussions. Each of the primary-school-aged children were accompanied by a caregiver which also changed the dynamics of the focus group. It is important that the facilitation of such groups takes into account the age of the participants and group dynamics (44), and that all participants are given the opportunity to contribute.

One factor that should be borne in mind is that the participants in our focus groups may not represent all backgrounds because they were self-selecting. It is possible that their issues may not be representative of the entire population. However, as difficulties with communication in noisy environments, listening effort and mismatch between sounds in the two ears are well reported in the literature they form reasonable goals for the BEARS training suite. It is possible that the focus groups were made up of young people with a particular interest in virtual reality training games. The future clinical trial to evaluate the effectiveness of the BEARS training suite will enroll young people from a wide range of backgrounds and interests to fully understand the value of the intervention.

## Data Availability Statement

The original contributions presented in the study are included in the article/supplementary material, further inquiries can be directed to the corresponding author/s.

## Ethics Statement

The studies involving human participants were reviewed and approved by University of Cambridge, Psychology Panel. Written informed consent to participate in this study was provided by the participants' legal guardian/next of kin.

## Author Contributions

DV, LP, DJ, and MM: conceptualization and methodology. DV: draft article writing. LP, MS-C, and MM: review and editing. MS-C, SD, CR, GD, and DJ: supported focus groups. SD, CR, and DJ: ran recruitment campaigns for both patients and clinicians. GD: project managed the workshops and participant groups. LP, YL, KS, and JA: engineering team who implemented changes and demonstrated concepts at workshops. BP: facilitated workshops and created prioritized action plans. MM, FE, NV, and DV: built the logic model through discussion with clinicians and exploration of the literature. All members of the team were involved in intellectual discussions following each phase and cycle of the project.

## Funding

This project was funded by NIHR PGfAR (201608). DV and MS-C salaries were funded by a Medical Research Council Senior Fellowship in Hearing (MR/2002537/1).

## Author Disclaimer

The views expressed are those of the authors and not necessarily those of the NIHR or the Department of Health and Social Care.

## Conflict of Interest

The authors declare that the research was conducted in the absence of any commercial or financial relationships that could be construed as a potential conflict of interest.

## Publisher's Note

All claims expressed in this article are solely those of the authors and do not necessarily represent those of their affiliated organizations, or those of the publisher, the editors and the reviewers. Any product that may be evaluated in this article, or claim that may be made by its manufacturer, is not guaranteed or endorsed by the publisher.

## References

[1] Hallewell M Salanitri D D'Cruz M Cobb S Picinali L Frost E. Dartanan: prototype evaluations of a serious game to engage children in the calibration of their hearing aid functionalities. J Rehabil Assist Technol Eng. (2021) 8:20556683211021527. 10.1177/20556683211021527 34290881 PMC8274128

[2] Hakobyan L Lumsden J O'Sullivan D. Participatory design: How to engage older adults in participatory design activities. Inter J Mobile Human Comp Inter. (2015) 7:78–92.

[3] Blauert J. Spatial Hearing: The Psychophysics of Human Sound Localization. Cambridge, Mass: MIT Press (1997). 10.7551/mitpress/6391.001.0001

[4] Kobler S Rosenhall U. Horizontal localization and speech intelligibility with bilateral and unilateral hearing aid amplification. Int J Audiol. (2002) 41:395–400. 10.3109/14992020209090416 12403607

[5] McAlpine D Jiang D Palmer AR. A neural code for low-frequency sound localization in mammals. Nat Neurosci. (2001) 4:396–401. 10.1038/86049 11276230

[6] Noble W Gatehouse S. Effects of bilateral versus unilateral hearing aid fitting on abilities measured by the speech, spatial, and qualities of hearing scale (SSQ). Int J Audiol. (2006) 45:172–81. 10.1080/14992020500376933 16579492

[7] Bronkhorst AW. The cocktail-party problem revisited: early processing and selection of multi-talker speech. Attent Percept Psychophys. (2015) 77:1465–87. 10.3758/s13414-015-0882-9 25828463 PMC4469089

[8] Nabelek AK Pickett JM. Monaural and binaural speech perception through hearing aids under noise and reverberation with normal and hearing-impaired listeners. J Speech Hear Res. (1974) 17:724–39. 10.1044/jshr.1704.724 4444292

[9] McArdle RA Killion M Mennite MA Chisolm TH. Are two ears not better than one? J Am Acad Audiol. (2012) 23:171–81. 10.3766/jaaa,.23.3.4 22436115

[10] Sarant J Harris D Bennet L Bant S. Bilateral versus unilateral cochlear implants in children: a study of spoken language outcomes. Ear Hear. (2014) 35:396–409. 10.1097/AUD.0000000000000022 24557003 PMC4072444

[11] Boons T Brokx JP Frijns JH Peeraer L Philips B Vermeulen A. Effect of pediatric bilateral cochlear implantation on language development. Arch Pediatr Adolesc Med. (2012) 166:28–34. 10.1001/archpediatrics.2011.748 22213747

[12] Sparreboom M Langereis MC Snik AF Mylanus EA. Long-term outcomes on spatial hearing, speech recognition and receptive vocabulary after sequential bilateral cochlear implantation in children. Res Dev Disabil. (2015) 36C:328–37. 10.1016/j.ridd.2014.10.030 25462493

[13] Lovett RE Vickers DA Summerfield AQ. Bilateral cochlear implantation for hearing-impaired children: criterion of candidacy derived from an observational study. Ear Hear. (2015) 36:14–23. 10.1097/AUD.0000000000000087 25170781

[14] Litovsky RY. Review of recent work on spatial hearing skills in children with bilateral cochlear implants. Cochlear Implants Int. (2011) 12(Supp. 1):S30–4. 10.1179/146701011X13001035752372 21756469 PMC3527898

[15] Grieco-Calub TM Litovsky RY. Sound localization skills in children who use bilateral cochlear implants and in children with normal acoustic hearing. Ear Hear. (2010) 31:645. 10.1097/AUD.0b013e3181e50a1d 20592615 PMC2932831

[16] Zheng Y Godar SP Litovsky RY. Development of sound localization strategies in children with bilateral cochlear implants. PLoS ONE. (2015) 10:e0135790. 10.1371/journal.pone.0135790 26288142 PMC4545829

[17] Hughes KC Galvin KL. Measuring listening effort expended by adolescents and young adults with unilateral or bilateral cochlear implants or normal hearing. Cochlear Implants Int. (2013) 14:121–9. 10.1179/1754762812Y.0000000009 23540588

[18] Lammers MJ van der Heijden GJ Pourier VE Grolman W. Bilateral cochlear implantation in children: a systematic review and best-evidence synthesis. Laryngoscope. (2014) 124:1694–9. 10.1002/lary.24582 24390811

[19] Firszt JB Reeder RM Dwyer NY Burton H Holden LK. Training results in individuals with unilateral severe to profound hearing loss. Hear Res. (2015) 319:48–55. 10.1016/j.heares.2014.11.005 25457655 PMC4291285

[20] Keating P Rosenior-Patten O Dahmen JC Bell O King A. Behavioral training promotes multiple adaptive processes following acute hearing loss. Elife. (2016) 5:e12264. 10.7554/eLife.12264 27008181 PMC4841776

[21] Keating P Johannes C Dahmen A King J. Context-specific reweighting of auditory spatial cues following altered experience during development. Curr Biol. (2013) 23:1291–9. 10.1016/j.cub.2013.05.045 23810532 PMC3722484

[22] Stitt P Picinali L Katz B. Auditory accommodation to poorly matched non-individual spectral localization cues through active learning. Sci Rep. (2019) 9:1–14. 10.1038/s41598-018-37873-0 30705332 PMC6355836

[23] Steadman M Kim C Lestang JH Goodman D Picinali L. Short-term effects of sound localization training in virtual reality. Sci Rep. (2019) 9:1–17. 10.1038/s41598-019-54811-w 31798004 PMC6893038

[24] Carlile S. The plastic ear and perceptual relearning in auditory spatial perception. Front Neurosci. (2014) 8:237. 10.3389/fnins.2014.00237 25147497 PMC4123622

[25] Trapeau R Aubrais V Schönwiesner M. Fast and persistent adaptation to new spectral cues for sound localization suggests a many-to-one mapping mechanism. J Acoust Soc Am. (2016) 140:879–90. 10.1121/1.4960568 27586720

[26] Watson CJ Carlile S Kelly H Balachandar K. Generalization of auditory accommodation to altered spectral cues. Sci Rep. (2017) 7:11588. 10.1038/s41598-017-11981-9 28912440 PMC5599623

[27] Ingvalson EM Lee B Fiebig P Wong PC. The effects of short-term computerized speech-in-noise training on postlingually deafened adult cochlear implant recipients. J Speech Lang Hear Res. (2013) 56:81–8. 10.1044/1092-4388(2012/11-0291) 22744139 PMC6771930

[28] Oba SI Fu QJ Galvin JJ III. Digit training in noise can improve cochlear implant users' speech understanding in noise. Ear Hear. (2011) 32:573–81. 10.1097/AUD.0b013e31820fc821 21389857 PMC3129451

[29] Zhang T Dorman MF Fu QJ Spahr AJ. Auditory training in patients with unilateral cochlear implant and contralateral acoustic stimulation. Ear Hear. (2012) 33:e70–9. 10.1097/AUD.0b013e318259e5dd 22622705 PMC3463714

[30] Casserly ED Barney EC. Auditory training with multiple talkers and passage-based semantic cohesion. J Speech Lang Hear Res. (2017) 60:159–71. 10.1044/2016_JSLHR-H-15-0357 28002542

[31] Green T Faulkner A Rosen S. Computer-based connected-text training of speech-in-noise perception for cochlear implant users. Trends Hear. (2019) 23:2331216519843878. 10.1177/2331216519843878 31010386 PMC6480987

[32] Cai Y Chen G Zhong X Yu G Mo H Jiang J. Influence of audiovisual training on horizontal sound localization and its related erp response. Front Hum Neurosci. (2018) 12:423. 10.3389/fnhum.2018.00423 30405377 PMC6206041

[33] Rayes H Al-Malky G Vickers D. Systematic review of auditory training in pediatric cochlear implant recipients. J Speech Lang Hear Res. (2019) 21:1574–93. 10.1044/2019_JSLHR-H-18-0252 31039327

[34] Whitton JP Hancock KE Shannon JM Polley DB. Audiomotor perceptual training enhances speech intelligibility in background noise. Curr Biol. (2017) 27:3237–47. 10.1016/j.cub.2017.09.014 29056453 PMC5997394

[35] Lewin K. Action research and minority problems. J Soc Issues. (1946) 2:34–46. 10.1111/j.1540-4560.1946.tb02295.x

[36] Reason P Heron J. Co-operative inquiry. In: Rethinking in Psychology. Harre R Smith J Van Langenhove L editors. London: Sage (1995). p. 122–42. 10.4135/9781446221792.n9

[37] Cuevas-Rodríguez M Picinali L González-Toledo D Garre C de la Rubia-Cuestas E Molina-Tanco L. 3D Tune-in toolkit: an open-source library for real-time binaural spatialisation. PLoS ONE. (2019) 14:e0211899. 10.1371/journal.pone.0211899 30856198 PMC6411112

[38] MRC-NIHR (36). Developing and Evaluating Complex Interventions. Available online at: http://www.mrc.ac.uk/complexinterventionsguidance (accessed July 12, 2021).

[39] Insel C Charifson M Somerville LH. Neurodevelopmental shifts in learned value transfer on cognitive control during adolescence. Dev Cogn Neurosci. (2019) 40:100730. 10.1016/j.dcn.2019.100730 31756586 PMC6934050

[40] Hallewell M Patel H Salanitri D Picinali L Cobb S Velzen J. Play and tune: user feedback in the development of a serious game for optimizing hearing aid orientation. Ergon Des. (2021) 29:14–24. 10.1177/1064804619899558

[41] Hanssen GK Dahl Y. A participatory design approach to develop an interactive sound environment simulator. Am J Audiol. (2016) 25:268–71. 10.1044/2016_AJA-16-0005 27768186

[42] Ferguson M Leighton P Brandreth M Wharrad H. Development of a multimedia educational programme for first-time hearing aid users: a participatory design. Int J Audiol. (2018) 57:600–9. 10.1080/14992027.2018.1457803 29718733

[43] Frost E Porat T Malhotra P Picinali L. (2020). A novel auditory-cognitive training app for delaying or preventing the onset of dementia: participatory design with stakeholders. JMIR Hum Fact. (1980) 7:e19880. 10.2196/19880 32996884 PMC7557448

[44] Adler K Salanterä S Zumstein-Shaha M. Focus group interviews in child, youth, and parent research: an integrative literature review. Int J Qual Res. (2019) 18:1–15. 10.1177/1609406919887274

